# The impact of modifying 3D printing parameters on mechanical strength and physical properties in vat photopolymerisation

**DOI:** 10.1038/s41598-025-97294-8

**Published:** 2025-04-12

**Authors:** Alice M G Cheadle, Eva Maier, William M Palin, Phillip L Tomson, Gowsihan Poologasundarampillai, Mohammed A Hadis

**Affiliations:** 1 Department of Dentistry, Dental and Biomaterial Science, College of Medicine and Health, University of Birmingham, Birmingham, UK; 2 Operative Dentistry and Periodontology, University Hospital Erlangen, Friedrich-Alexander-University Erlangen-Nuremberg, Erlangen, Germany

**Keywords:** Vat photopolymerisation, Build orientation, Tensile strength, Degree of conversion, Additive manufacture, Anisotropy, Design, synthesis and processing, Electronic devices, Characterization and analytical techniques

## Abstract

Vat photopolymerisation is frequently used to produce parts through additive manufacture by way of layer-by-layer resin polymerisation. A post curing process is often used to ensure optimised polymerisation of the printed structures. The part to be printed is digitally orientated in relation to the principal building axis and build platform. The effects of part (or object) print orientation, post-cure time / temperature on mechanical and physical properties were investigated. Scaled ASTM D638 dumbbell shaped specimens were printed to measure mechanical properties and degree of conversion; printed cylindrical discs (15 mm diameter, 1.6 mm thick) were printed to evaluate colour change and UV-absorbance. Dumbbell shaped specimens with principal axis perpendicular to the build platform had significantly better tensile strength (*p* < 0.05) and degree of conversion (*p* < 0.05). Increased post-cure temperature and time significantly influenced degree of conversion, yellowing of samples and tensile strength (*p* < 0.05). This study highlights the importance of optimising both orientation of build and post-cure parameters, as well as potential to exploit post-cure protocols to overcome anisotropy, which is of great clinical importance in applications such as dental restorations, bone scaffolds and prostheses. Knowledge of how to optimise physical and mechanical properties of SLA-printed parts in the context of the application is essential for anyone directly or indirectly employing the technology.

## Introduction

In an increasingly digital age, where computer aided design and manufacture is commonplace with hobbyists and industry, additive manufacturing has developed into an accessible method of fabrication of objects with specific properties, such as heat resistance, flexibility and high toughness. Critically, since the expiry of patents which prevented companies from exploiting the approach^1^, additive manufacture is now a widely attainable and affordable technology. The two most common processes for vat photopolymerisation are stereolithography (SLA) and digital light processing (DLP), the former being developed in the 1980s^2^. SLA printers utilise a vat photopolymerisation process, where the liquid resin is filled in a reservoir with a transparent foil at the base. Using a laser (usually, 380–420 nm, < 250 mW power) the photopolymer is cured layer-by-layer onto a build platform, rastering across the x-y axis plane until a layer is complete, before moving in the z-axis to incrementally build up the layers that are defined in the 3D model data^3^. Uncured resin remains in the tank after completion of a print which can be used for another print and thus is less wasteful than subtractive manufacturing processes. Modern printers will allow for selection of z-layer height, position and orientation with respect to the build platform (printing direction) via dedicated Computer-Aided-Design (CAD) software which influences print duration and might affect material properties^4^.

During printing, an exposure of photonic energy over a specified amount of time triggers polymerisation, resulting in the conversion of a finite thickness of photocurable resin into polymer (typically 25–100 μm; defined in the 3D print file as the layer thickness) and width (limited by the laser beam width, usually < 150 μm)^5^. Variation of the incident radiation will affect exposure and thus cure depth^6,7^. Photocurable resins are cured by way of a free radical addition polymerisation. Typically, laser light is absorbed by a Type I photoinitiator, generating free radicals that initiate the polymerisation reaction. When the laser is off, radicals cease to be generated through light absorption, whilst radical-radical terminations continue resulting in a curing plateau. Vat photopolymerisation resins also include radical inhibitors and other additives that enhance the effects of oxygen inhibition (to prevent unwanted cure in deeper layers of the resin reservoir), which further impact cure kinetics. Printed parts are thus produced in an incompletely polymerised state (so-called ‘green state’) which is then post-cured with ‘floodlight’ systems to ensure further polymerisation^5,8,9^. Post-curing devices can be temperature controlled and are usually equipped with light sources delivering the same wavelength of light as the printer^10^. Temperature (usually up to 80 °C, or well below the glass transition temperature of the polymer) and time of the post-cure protocol are set by the operator as recommended by the resin and printer manufacturer. Recommended post-cure parameters are designed to provide optimum outcomes under ideal conditions, and do not consider print variables. Therefore, for functional SLA-printed parts a one size fits all approach may not provide optimal parameters; guidelines and limits on ideal post-cure parameters should be established.

‘Closed system’ SLA printers will have predetermined settings for their proprietary resins. These may include resin temperature, angle / intensity of light exposure, exposure time and laser spot size. In printers with ‘open systems’, further settings can be adjusted, for example exposure time and irradiance. Most users of SLA printers will purchase pre-prepared resins which are designed to optimise specific properties for any given application. Although manufacturers’ information and Safety Data Sheets usually offer restricted insight into resin composition, pigments, dyes, inhibitors and photoinitiators will vary. Furthermore, specific details are often not disclosed to protect intellectual property of the developer, making it difficult for even experienced users to tailor optimised mechanical and physical properties through printing protocols.

Many parameters for SLA printing including those under operator control can affect the physical and mechanical properties of the printed products. These parameters may affect layer thickness, dimensional accuracy, and the degree of polymer conversion^11–13^. Orientation and therefore direction of the print can affect mechanical strength with respect to load direction^14,15^ and dimensional accuracy^16^. Variability in post-curing protocol from type of equipment used^10,17^ time and temperature of cure can affect colour, mechanical strength and hardness of the printed product^18,19^. Many studies have assessed mechanical strength by way of flexural strength testing^4,14,18,20^. Flexural testing is commonly used for testing materials with a brittle nature. Printed parts using unfilled resins are likely to exhibit more flexibility and thus tensile testing becomes a more appropriate mechanical test than flexion^21^.

Printed parts, particularly in the green state, exhibit anisotropy; Monzón et al.^22^ tested mechnical strength under flexural and tensile loading, although two of the three resins investigated were casting resins where anisotropy may not be relevant as they are not used in load-bearing areas but just for modelling. For example, a printed ornamental model would not need careful consideration of anisotropy but a critical component of an industrial machine would^3^. Monzón et al.^22^ concluded that build direction had a significant effect on mechanical properties, particlarly when the post-curing process did not occur. This study hypothesised that varying post-cure protocol could help reduce anisotropy because of sufficient polymerisation, allowing better mechanical properties. Previous studies that have investigated anisotropy that have tested tensile strength have employed ISO-Standard 527-2^22–25^ which has been widely used for injection-moulded and extruded plastic parts; the choice of tensile specimen needs careful consideration.

Colour stability of 3D printed materials is essential for some applications, such as those used to print dental restorations where tooth colour mimicry are critical. Colour change of dental resins has previously been linked oxidative by-products. Two photoinitiators commonly used in vat photopolymerisation materials are (diphenyl (2,4,6-trimethylbenzoyl) phosphine oxide (TPO) and bis phenyl (2,4,6-trimethylbenzoyl) phosphine oxide (BAPO); yellowing of resins was reported with TPO at increased curing temperatures^26^. Within 3D printing, colour change of resins during the printing process could be due to many factors which have not been fully explored. Colour change has been reported due to post-cure protocol^18,27^, translucency of 3D printing resins and print orientation^28^. In this study, we introduce UV spectroscopy as a novel analytical tool to quantitively assess changes in the optical properties of printed resins to provide novel insight into the mechanisms driving colour change.

Understanding the effects of printing parameters on the properties of printed parts is essential due to the variety of available printers, post-curing devices and materials^12^.

The aim of this study was to evaluate the effect of orientation of print and post-cure protocol on mechanical and physical properties of printed parts using Clear V4 resin in the Form 2 SLA printer and FormCure post-cure device (FormLab, Sommerville, USA).

## Materials and methods

### Tensile testing

**Fig. 1:**
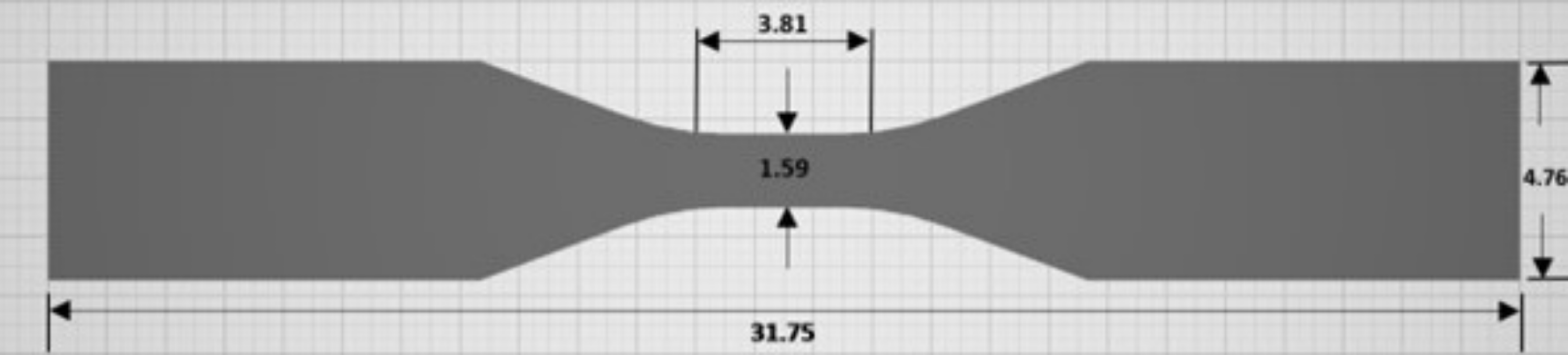
ASTM D638 Type V tensile specimens at 0.5 scale were sketched using CAD software (Fusion 360, Autodesk). Dimensions in mm. Specimen thickness 1.6 mm. Post-print dimension averages: width of gauge 1.59 mm (± 0.05) and thickness 1.62 mm (± 0.061).

**Fig. 2:**
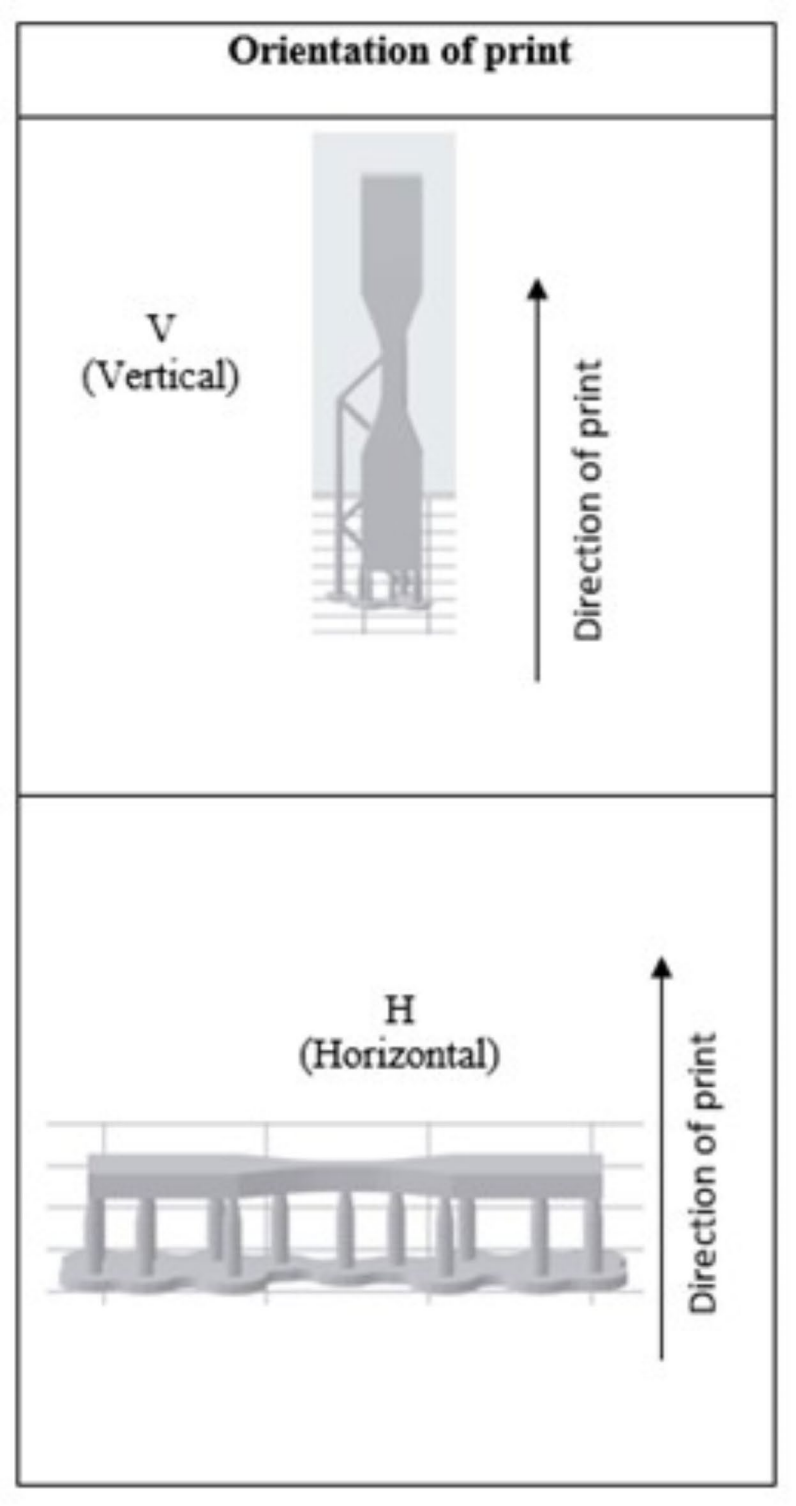
Orientation of the tensile bars were either printed horizontally or vertically with relation to the build platform. The two other variables were time of post-cure (0, 5, 10, 15, 30, 60–90 min) and temperature of post-cure (35, 60–80 °C) with 38 groups in total including the ‘no-post cure group’ which has no exposure to heat. Groups were designated according to print orientation, post cure temperature and post cure exposure time (e.g. print orientation: horizontal, post cure temperature: 35 degrees, post cure exposure time 30 min is designated as H 35 °C 30 m.

Dumbbell shaped specimens were designed using computer aided design (CAD) software (Fusion 360, Autodesk, San Francisco, USA) according to the ASTM D638 standard (Fig. 1). The file was saved as a standard tessellation language (STL) file using Fusion 360 default settings, which produced a medium resolution triangle mesh with 92 triangles, suitable for 3D printing. The STL file was exported to the 3D printing software programme (PreForm, Formlab, Sommerville, USA) where specimens were standardised in their 3D printing orientations (Fig. 2) and support structures added. Specimens were then printed with commercially available resin (Clear V4, FormLab, Sommerville, USA) using an SLA printer (Form 2, Formlabs, Somerville, USA), in two relative print directions: vertical and horizontal (Fig. 2). Horizontally printed ASTM D638 tensile specimen bar had its long axis and layers parallel to the build platform, the supporting sprues perpendicular to the build platform. Vertically printed ASTM D638 tensile specimen bar had its long axis and layers perpendicular to the build platform. Following printing, specimens were post-cured in a post-cure device (FormCure, Formlabs, Somerville, USA) using various temperatures (35, 60–80 °C^)^ and exposure times (0, 5, 10, 15, 30, 60–90 min).

All samples were printed with a layer thickness of 100 μm using one batch of resin. Samples were washed and dried as per manufacturer’s instructions for the resin: a 10-minute wash in 99% isopropyl alcohol (Merck Life Science, Kent, UK) and air drying. Specimens were then cured for the respective time and temperature as given by the experimental protocol (Fig. 2), using a heat and light polymerisation unit (FormCure, Formlabs, Somerville, USA). Support structures were removed after post-cure as per manufacturer guidelines.

Ten specimens within each of the 38 groups were tested under tensile load using a universal testing machine (MTS, Minnesota, USA). A 5 kN load cell was used with a crosshead speed of 3 mm/min. Each specimen was loaded into metal tensile screw clamps and loaded until failure. The tensile strength was calculated using Eq. 1, as per ASTM D638:

1 $$\:\sigma\:=\frac{F}{A}\:\text{o}\text{r}\:Tensile\:Strength\left(MPa\right)=\frac{Force\:\left(N\right)}{Cross\:sectional\:area\:\left({mm}^{2}\right)}$$

Percentage elongation of the specimens before failure was calculated using the recorded extension of the specimen against the original gauge length of the tensile bar (Eq. 2).

2 $$\:\%\:elongation=\frac{extension\:\left(mm\right)}{gauge\:length\:\left(mm\right)}x\:100$$

### Degree of conversion

A sample of uncured liquid resin (Clear V4, FormLab) was placed in a mould of 100 μm thickness (equal to the printer layer thickness) and covered with a transparent cover slip placed carefully ensuring no air bubbles. Peak absorption was measured using Fourier Transform mid-infrared spectroscopy (FTIR ATR-mid IR, Nicolet 6700, Thermo Scientific, Pittsburgh, PA, USA). The peaks of the aliphatic C=C group (C=C; 1637 cm^− 1^) and the amide II (NH_2_; 1533 cm^− 1^) groups were identified on the spectrum for the UDMA based Clear V4 FormLab resin. The amide II was assigned as the isosbestic peak since this remained constant prior to and following cure. Polymerised specimens (*n* = 10) were produced as per those in the tensile testing methodology and degree of conversion was assessed. The central portion of the gauge length of the specimen was positioned on the ATR crystal, using the same orientation, alignment, measuring position and reproducible pressure for each reading. Respectively, degree of conversion was calculated using Eq. 3:

3 $$\:Conversion\:\%=1-\frac{aliphatic/isosbestic\:\left(peak\:areas\:of\:polymer\right)}{aliphatic/isosbestic\:\left(peak\:areas\:of\:monomer\right)} \times 100$$

### Colourimetry

Disc-shaped specimens were sketched for colourimetry using the same CAD software as for the tensile test specimens. Specimens were constructed 1.6 mm thick to match the tensile bar thickness, and with a diameter of 15 mm to fit the spectrophotometer platform (CM-2600d; Konica, Minolta, UK). Three specimens (*n* = 3) per group were printed and post-processed respectively as previously described (Fig. 2).

The spectrophotometer was used to measure the change in b* value using the CIELAB scale (*Commission International de l’èclairage*: L*a*b*) which identifies the perceptual lightness (L*) and the amount of red, green, blue, and yellow (a* and b*) present in the specimen. An increase in b* value indicates yellowing of the specimen, which was the interesting marker for discolouration in the current project. Prior to testing each group, the spectrophotometer was calibrated using the ‘white calibration plate’ provided by the instrument manufacturer. Colour measurements were taken of the uncured resin and then repeated for each of the samples in the varying post-cure protocol groups.

### UV spectroscopy

Specimen absorbance was measured through the gauge of tensile bars from each group (*n* = 5) using a UV-vis spectrometer (USB400, Ocean Optics). The spectrometer was set-up so that bare 1000 μm fibres were held in a custom-made jig; one fibre was connected to a tungsten halogen light source (HL200, Ocean Insight) - used to emit UV-Visible-NIR wavelengths (360–2400 nm), and the other connected to the spectrometer and OceanView application software (Ocean Optics, Duiven, The Netherlands).

### Statistical analysis

General Linear Model of all independent variables (time and temperature of post-cure and orientation of print) was carried out, as well as complimentary separate one-way ANOVAs and post-hoc Tukey comparisons (*p* < 0.05).

## Results

### Tensile testing

**Fig. 3:**
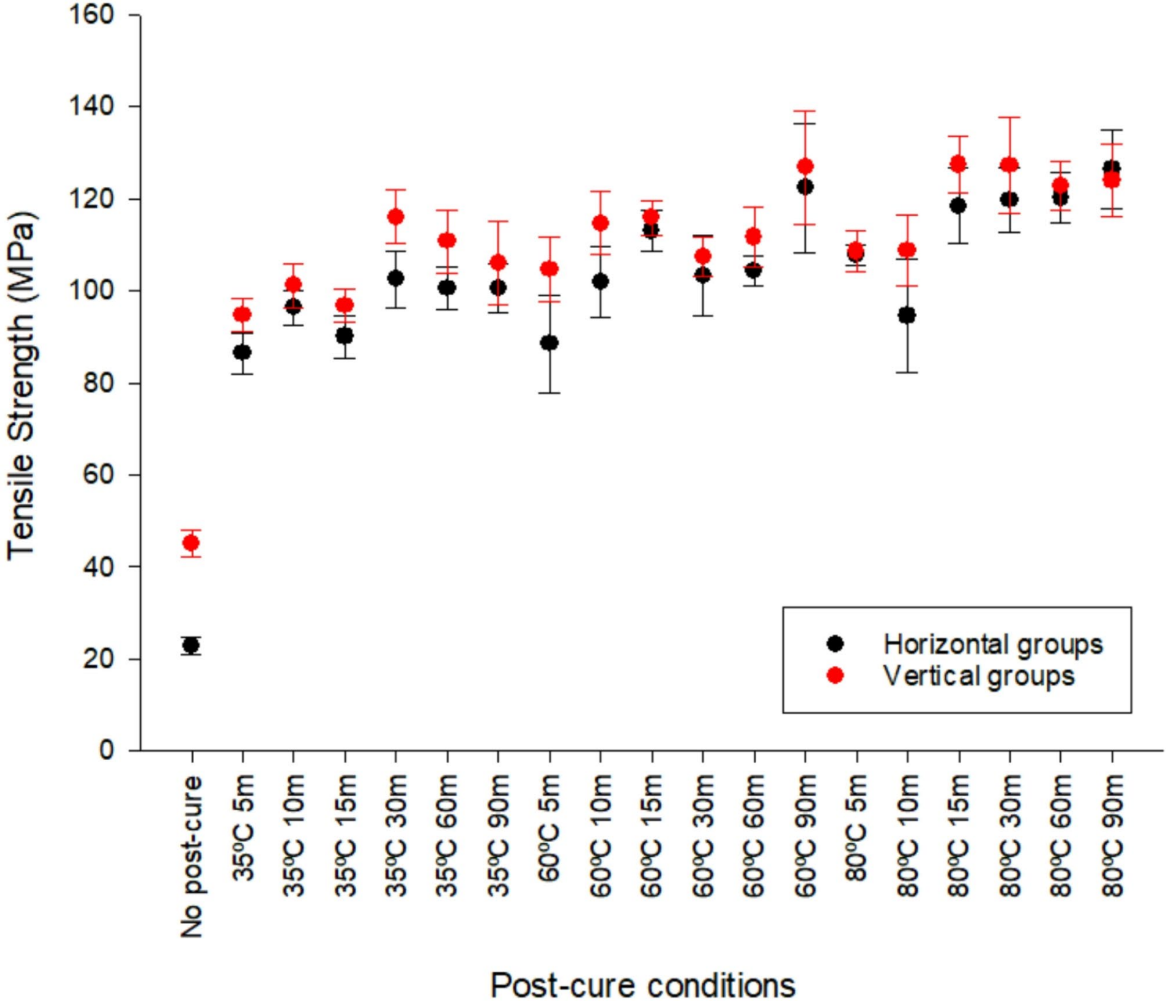
Average tensile strength (MPa) of all groups (*n* = 10). The group with the highest average tensile strength was V 80ºC 15 min (127.37 MPa ± 6.19) and the lowest was H no post-cure (22.84 MPa ± 1.92).

**Fig. 4:**
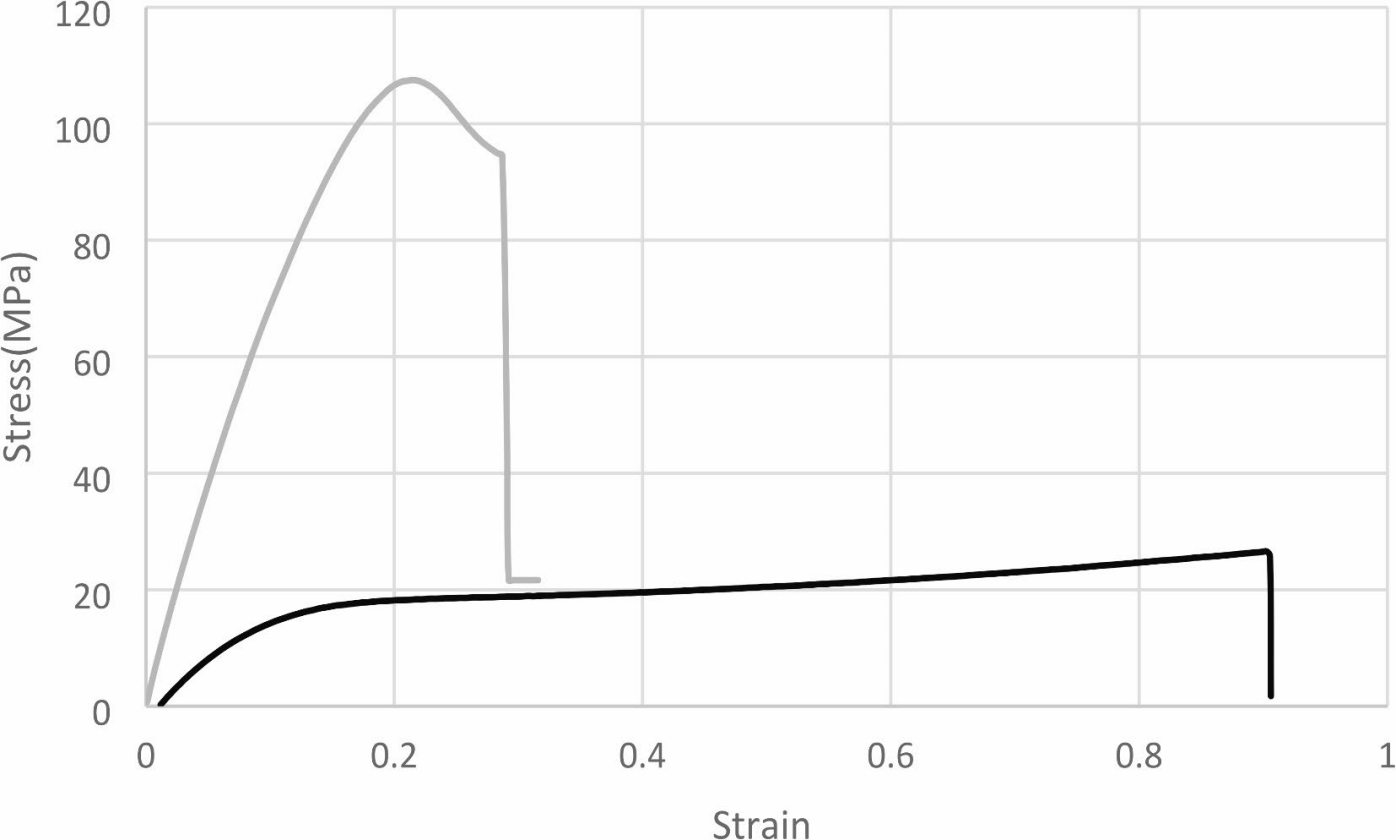
Graph of two example stress-strain curves for distinctly different post-cure conditions: V 60 °C 30 min (grey) and H no post-cure (black).

**Fig. 5:**
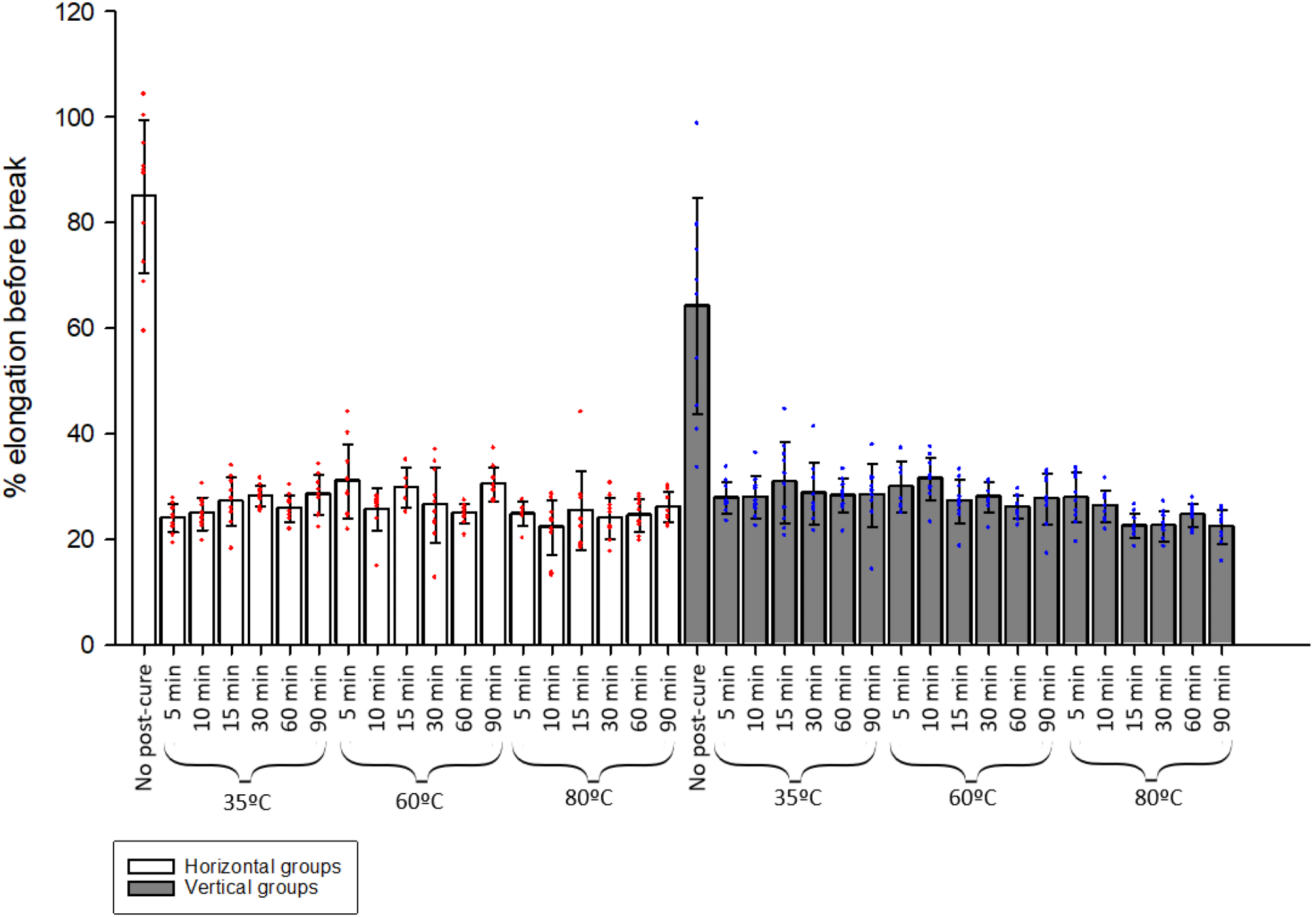
Bar chart showing % elongation of specimens before break, bars depicting the group average with standard deviation and points for individual specimens. Increased post-cure time showed significant (*p* < 0.05) decrease in elongation before break in four out of six temperature groups (H35 ºC, H60 ºC, V60 ºC and V80 ºC), likewise for an increase in temperature in all vertical time groups (apart from V5mins), but only two of the horizontal time groups (H5mins and H90mins).

Printed tensile bars were tested to failure to assess the effect of orientation of print and post-cure conditions on mechanical strength (Fig. 3). There were significant differences (*p* < 0.001) in tensile strength where temperature (df = 2; F = 108.06) > orientation (df = 1; F = 61.07) > time (df = 5 F = 36.94). There was significantly higher tensile strength in vertically orientated specimens (*p* < 0.001). Tensile strength increased with post-cure exposure time (*p* < 0.05) and higher curing temperatures (*p* < 0.001); the highest tensile strengths were those of the 80℃ groups (*p* < 0.05),

Percentage elongation of tensile bar length before break highlighted a more elastic nature of green state specimens, lengthening significantly more (*p* < 0.001) before failure than those that underwent post-processing (Fig. 4). Furthermore, green state horizontal bars were subject to 84% (± 14.4) elongation before failure compared to green state vertical bars (64% (± 20.4)). However, this anisotropy was overcome by post-cure processing where orientation in post-cured groups did not significantly impact percentage elongation before break (Fig. 5).

### Degree of conversion

**Fig. 6:**
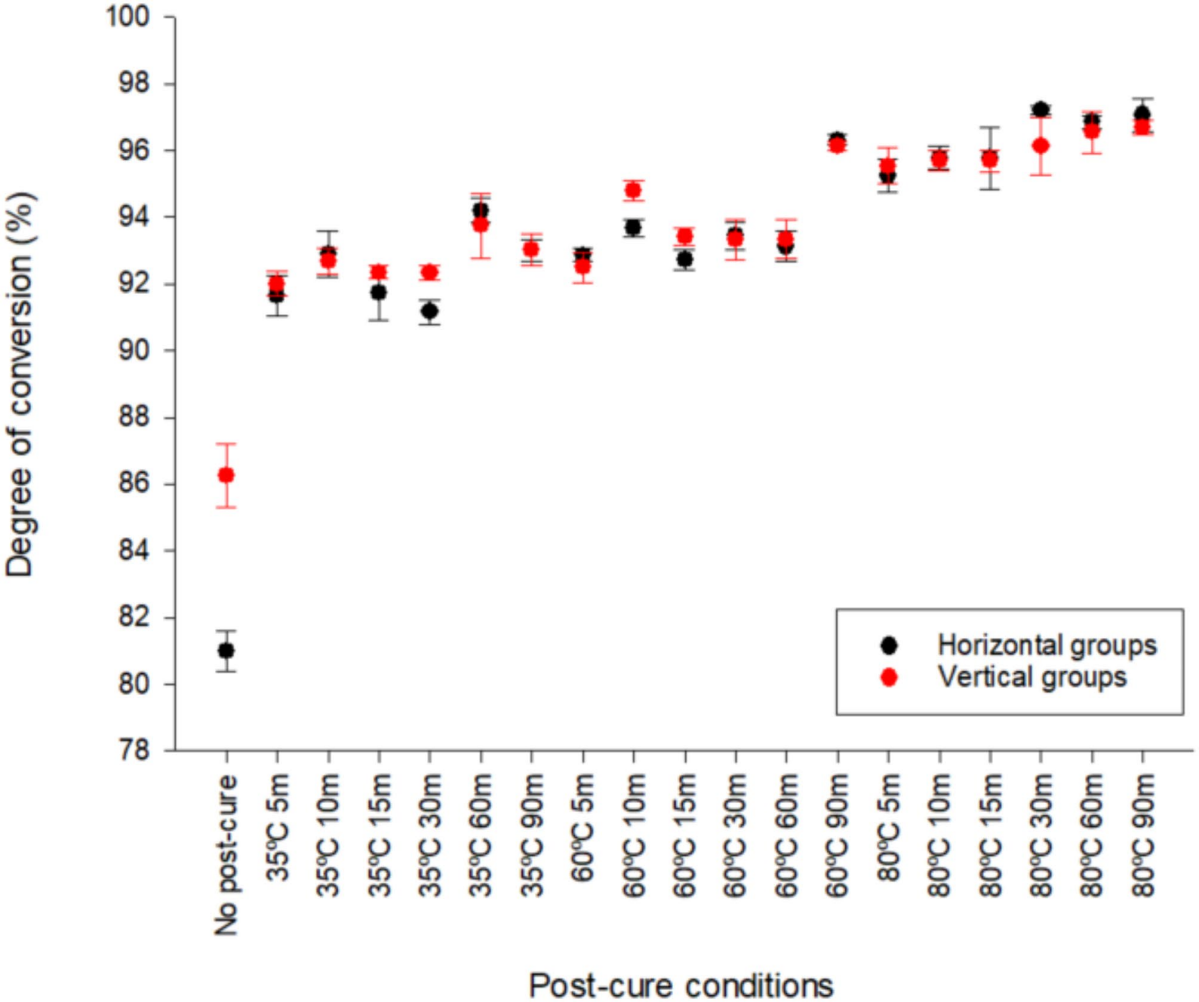
Average degree of conversion (%) of all groups (*n* = 10). The group with the highest degree of conversion (*p* < 0.05) was H80°C 30 min (97.20% +/- 0.13) and the lowest was H no post-cure (80.99% +/- 0.61).

Degree of conversion data was gathered to assess the effect of orientation and post-cure processes on polymerisation. There were significant differences (*p* < 0.001) in degree of conversion where temperature (df = 2; F = 648.56) > time (df = 5; F = 53.02) (Fig. 6). There was no statistically significant difference in orientation (df = 1; F = 0.53). There was significantly higher degree of conversion in samples cured for 90 min cf. 5 min, irrespective of orientation and temperature (*p* < 0.05). There was significant difference in degree of conversion where 80 °C > 60 °C > 35 °C in all groups apart from H&V60min and V5min. There was significant difference in degree of conversion where 80 °C > 35 °C in all groups (*p* < 0.005). Average degree of conversion for horizontal no post-cure was half that of the vertical no post-cure (22.84 MPa ± 1.92 cf. 44.99 MPa ± 2.84).

### Colourimetry

**Fig. 7:**
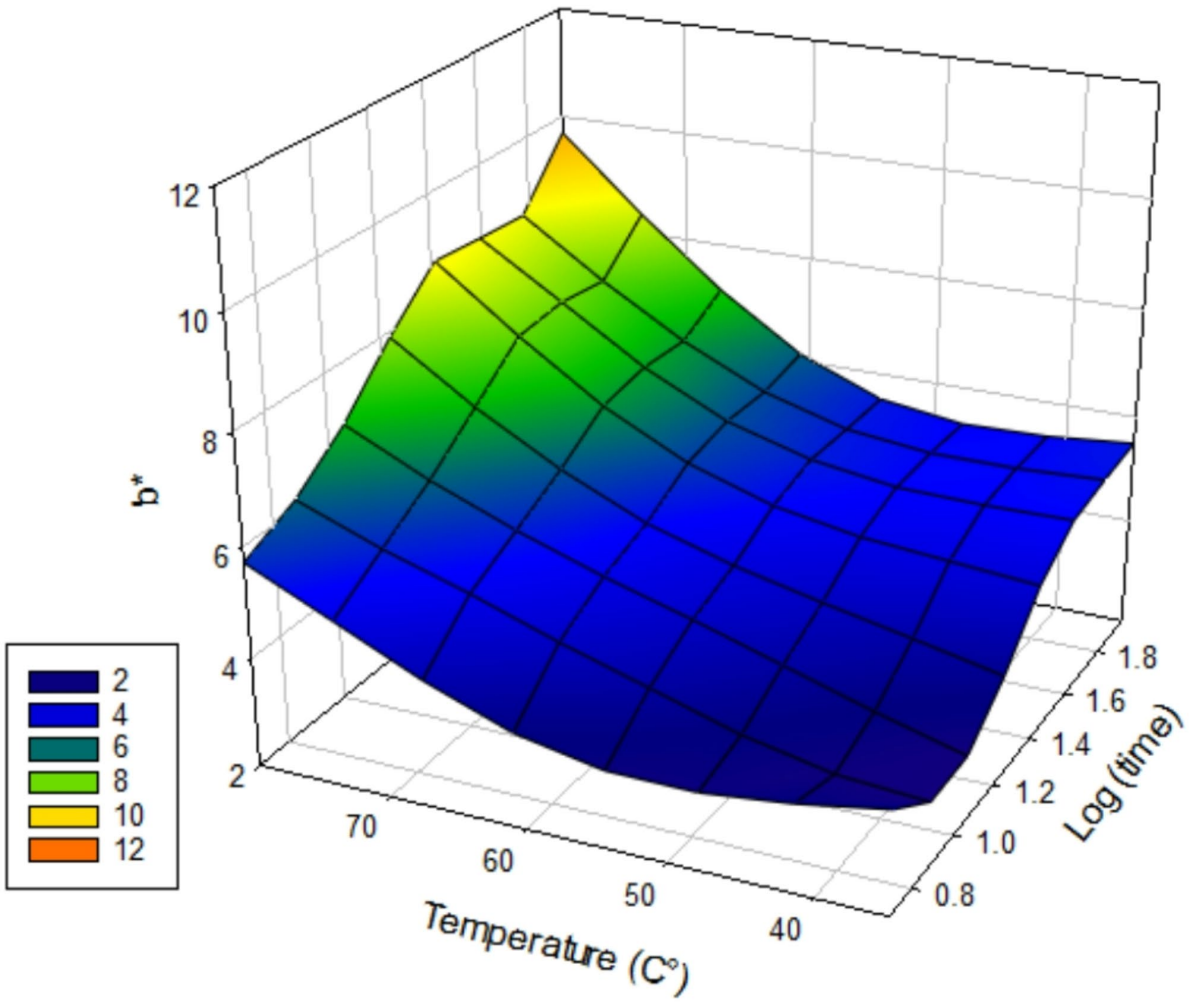
Surface response curve showing average post-cure b* values for vertical groups of log(time) vs. temperature (℃) vs. b* (*n* = 3).

**Fig. 8:**
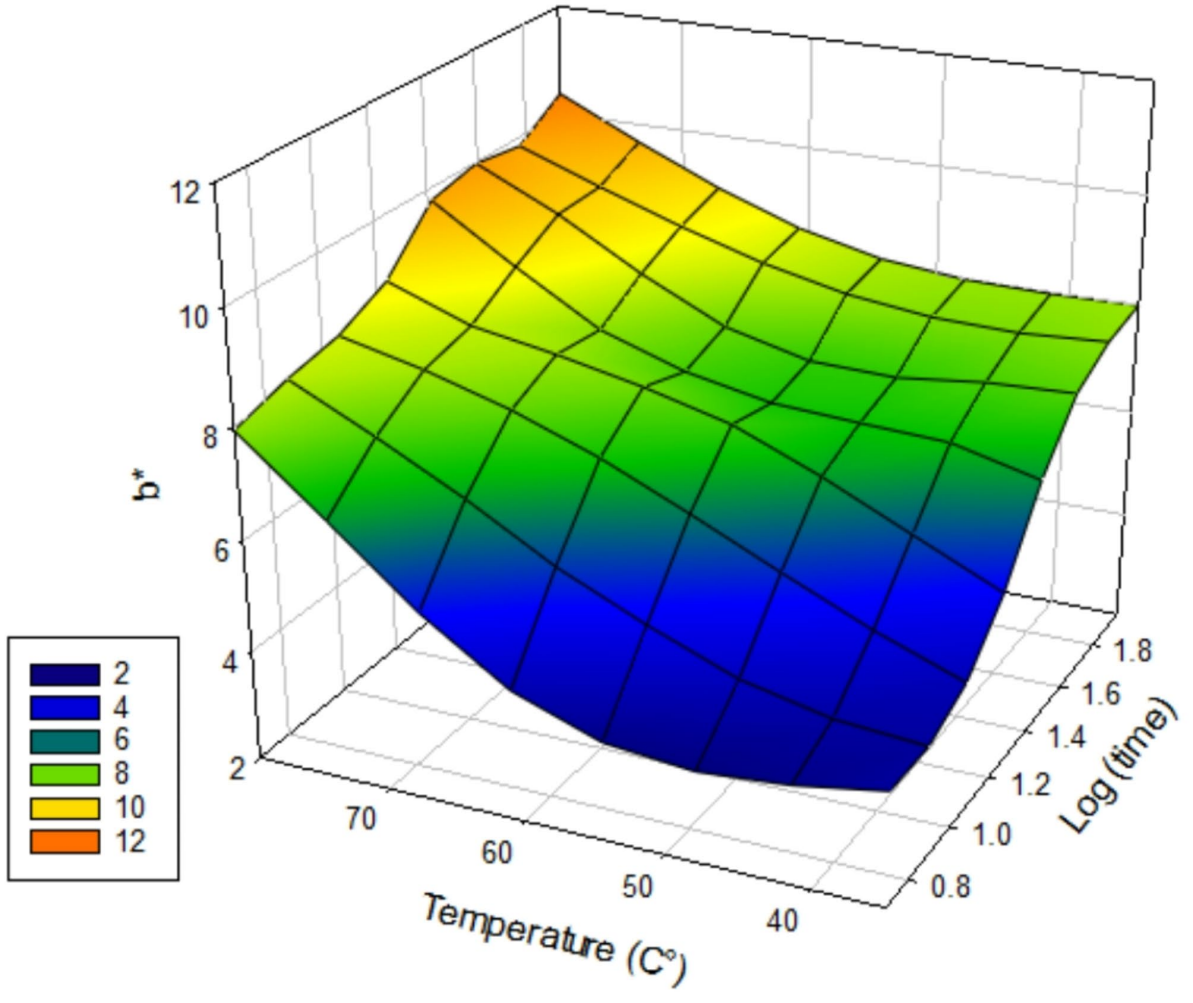
Surface response curve showing average post-cure b* values for horizontal groups of log(time) vs. temperature (℃) vs. b*. There is a greater effect of post-cure on the horizontal specimens, indicated by a reduction in the blue area when comparing with Fig. 6a.

To evaluate discolouration caused by the post-cure processes, the change in b* value (indicating yellowing) was recorded using colourimetry data. Surface response curves were used to visualise the increase in average b* values in vertically (Fig. 7) and horizontally (Fig. 8) printed groups in relation to post-cure temperature and time. There were significant differences (*p* < 0.001) in post-cure b* value where temperature (df = 2; F = 301.64) > orientation (df = 1; F = 197.60) > time (df = 3; F = 79.25). Horizontally orientated groups had significantly higher post-cure b* values (more yellow) (*p* < 0.001). There were significantly higher post-cure b* values in samples cured for 90 min, 60 min and 30 min cf. 10 min and 5 min, irrespective of orientation (*p* < 0.05). There were significantly higher post-cure b* values in samples cured at 80 °C cf. 35 °C, irrespective of orientation (*p* < 0.05) and higher post-cure b* values in samples cured at 60℃ cf. 35℃ in 8/12 temperature groups (*p* < 0.05).The highest post-cure b* value was the H80°C 90 min group (10.38 +/- 0.37) and the lowest was V no post-cure (3.60 ± 0.11).

### UV spectroscopy

Specimen absorption was measured using absorption at 420 nm to quantitively assess changes in the colour of the printed resin (Table 1): There were significant differences (*p* < 0.005) in absorption at 420 nm where time (df = 3; F = 36.82) > orientation (df = 1; F = 8.51) > temperature (df = 2; F = 4.86). Significantly higher absorption in horizontally orientated specimens (*p* < 0.05) was seen in samples cured for 5 and 10 min cf. 60 min and 90 min, irrespective of orientation (*p* < 0.05). The lowest was V80°C 90 min (OD_420_ = 0.31 +/- 0.05), and the highest H80℃10 min (OD_420_ = 0.41 +/- 0.01).

**Table 1:** Average absorption at 420 nm peak (attributed to photopolymerisation by-products) through samples using UV spectroscopy with standard deviation in parentheses.

Groups	Absorption at 420 nm (OD)			
	Horizontal		Vertical	
No post-cure	0.38	(0.01)	0.37	(0.01)
35ºC / 5 min	0.39	(0.00)	0.39	(0.01)
35ºC / 10 min	0.39	(0.01)	0.38	(0.01)
35ºC / 15 min	0.40	(0.03)	0.40	(0.01)
35ºC / 30 min	0.39	(0.01)	0.38	(0.01)
35ºC/60 min	0.37	(0.01)	0.36	(0.01)
35ºC / 90 min	0.36	(0.02)	0.36	(0.01)
60ºC / 5 min	0.40	(0.02)	0.39	(0.02)
60ºC / 10 min	0.40	(0.01)	0.39	(0.010
60ºC / 15 min	0.39	(0.01)	0.39	(0.01)
60ºC / 30 min	0.38	(0.02)	0.37	(0.020
60ºC / 60 min	0.35	(0.02)	0.35	(0.02)
60ºC / 90 min	0.34	(0.01)	0.33	(0.02)
80ºC / 5 min	0.40	(0.02)	0.39	(0.01)
80ºC / 10 min	0.41	(0.01)	0.39	(0.04)
80ºC / 15 min	0.38	(0.01)	0.38	(0.05)
80ºC / 30 min	0.39	(0.01)	0.36	(0.01)
80ºC / 60 min	0.35	(0.02)	0.34	(0.02)
80ºC / 90 min	0.32	(0.01)	0.31	(0.05)

## Discussion

The aim of the current study was to evaluate the effect of post-cure parameters and printing orientation on mechanical and physical properties of an unfilled 3D printable resin. The current results showed that tensile strength, degree of conversion and colour can be significantly affected by print orientation and post-cure time and temperature. Manufacturers recommend a post-cure protocol suitable for specific resins, driven by a balance between the time required for manufacturing protocols and optimising mechanical properties. From a material research point of view, reference to an evidence-based range of possible post-cure conditions and information on print orientation might result in improved mechanical strength and physical properties, regardless of a potentially more time-consuming procedure.

### Effect of orientation

Tensile strength was significantly affected by the orientation of the printed specimens confirming anisotropy of the printed tensile bars. Mechanical properties are affected by the direction of build with vertically printed specimens (with their layers perpendicular to tensile strain) exhibiting better mechanical properties in both the present study under tensile testing and flexural testing^22^. The low brittleness of the unfilled resins tested here suggested that tensile testing was a more appropriate mechanical test. Adaptation of the ASTM D368 standard was made to perform tensile testing of specimen. A round robin method could be employed to develop standards specifically for additive manufacturing, particularly with regard to the variety of materials available for the same purpose and to study the effect of post-cure parameters on filled printable resins. ASTM D638 was chosen over ISO 527-2 owing to more favourable specimen geometry, testing parameters, strain localisation and strain rate for the tested resin.

Anisotropy can be explained by the stacking direction of layers as a result of layer-by-layer printing and their exposure during post-cure, improving crosslinking between layers^12^. The current SLA technology uses a rastering laser to cure liquid resin; typically, printers vary laser raster speed and/or power settings for the ‘border’ and ‘fill’ of the specimen, i.e. the vectors used to scan the cross-sectional area of the print^12^. Whilst a higher degree of conversion was not seen in (post-cured) vertically orientated specimens, the proportion of printing vectors used could help to explain anisotropy: border vectors operate at slower speeds, which allows for an increased radiant exposure (or fluence) in these regions and therefore a higher degree of polymerisation. For vertically printed specimens the proportion of the layers printed by border vectors is higher in comparison with the horizontal bars, and could contribute to their higher tensile strength. Furthermore, the resin in the present study was transparent, allowing more laser light transmission, and may explain the lack of impact of orientation on degree of conversion. Previous studies that use resins with a degree of opacity have identified orientation to have an impact degree of conversion^18^.

Although orientation did not significantly affect degree of conversion (F = 0.53), it was the second most important variable for tensile strength (F = 61.07), colour (F = 197.60) and UV absorption (F = 8.51). There was significantly higher degree of conversion of vertically printed specimens in the green state compared to horizontally printed specimens. However, following post cure processing this difference was no longer significant, which may be related to sufficient light and heat energy being able to conceal or nullify the differences in degree of conversion between orientations, despite differences in tensile strength still being apparent between print orientation. This could be demonstrated by degree of conversion measurements being taken only on the surface of the sample, should this be more affected by post cure than the interior of the sample. Colour and UV absorption still highlighted differences between orientation because of the transparency of the material and a resultant measurement perhaps more representative of the whole of the specimen. Future development could involve measuring degree of conversion in the deeper layers of specimens, this would be useful to understand the homogeneity of polymerisation but provides challenges in sectioning small incompletely cured specimens without artificially increasing degree of conversion by heat generation caused by sectioning.

### Effect of time and temperature

In the current study, post-cure temperature was the most significant variable influencing tensile strength, degree of conversion and colour. Time was the most significant variable for absorption at 420 nm peak through the samples, with longer times allowing more opportunity for photooxidation of TPO (and thus lower absorption of 420 nm). With respect to time of post-cure, the most significant increase in colour, degree of conversion and tensile strength was seen between the no-post-cure group and 5 min, confirming that post-cure is essential to achieve desirable mechanical and physical properties to allow for further polymerisation and crosslinking of polymer chains^5,8,9^.

Specimens cured at higher temperatures and with longer post-cure irradiation times had lower absorption at 420 nm as well as significantly higher post-cure b* values. Photooxidation causes polymer chains to break, releasing radicals which degrade the polymer and may cause colour change which is further exacerbated by heat (Hadis et al., 2012). By-products of oxidation may also cause discolouration. Rapid polymerisation of TPO and BAPO at higher temperatures may also cause the production of coloured peroxide resulting in yellowing^26^. Radicals and oxidative by-products might explain the significant difference in tensile strength should they vary in production between vertical and horizontal groups, with the latter exhibiting lower tensile strength. A higher production of oxidative by-products produced by breakdown of the polymer under prolonged light exposure may act as plasticisers and reduce tensile strength^26^. Tahayeri et al.^29^ found that degree of conversion of specimens printed closer to the build platform was higher; thus, the testing area (gauge) of the horizontally printed tensile bars would be closer to the build platform compared to vertical printed test pieces, and may experience more light exposure and by-product/radical formation leading to discolouration. For applications where colour is important, for example a provisional crown for dental restoration, it would be prudent to optimise curing conditions without compromising shade. The latest resin within the dental library of FormLab (Premium Teeth Resin) uses ethyl phenyl (2,4,6- trimethylbenzoyl) phosphinate (TPO-L) instead of TPO; in an application where shade is critical, this could be a change in order to improve colour stability.

An increase in post-cure temperature and time increases cross-linking and thus the degree of polymer conversion^30^. Although time impacted degree of conversion less than temperature, a considerably longer cure time of 90 min generally produced significantly higher degree of conversion and tensile strength compared to their counterpart groups of 5 min at respective higher temperatures (e.g. 35 °C 90 min cf. 60 °C 5 min). This suggested longer cure times can be used to compensate for lower post-curing temperatures. The increase in temperature of the curing oven does have practical considerations as the heating to 80 °C took 45 min. Despite this additional time to heat the oven, it may be better than increasing the time of post cure, as temperature had a greater effect than time on tensile strength, degree of conversion and colour. Consideration of the glass transition temperature is required with respect to the limit of the oven temperature.

The results of this study suggest that the anisotropy of the printed material was effectively mitigated by increasing post-cure time and temperature, promoting enhanced crosslinking and more uniform mechanical properties across print orientations which could have significant importance in some clinical applications including dental restorations, bone scaffolds and prostheses. Post-cure conditions and ideal mechanical properties would need to be balanced with any change to colour; some applications may require both - critical mechanical *and* aesthetic properties. For example, in dentistry where a printed crown needs to withstand the forces of mastication but be unacceptable with a yellow discolouration. The methods used here could be exploited by others to further study commercial and custom resins aligned to their respective applications using printers and post-cure devices and allow for development of evidence-based guidelines for printing to ensure printed objects are fit-for-purpose. Further research could investigate the knowledge base on printing and curing protocols of 3D printing resins, beyond ‘automated settings’, amongst prescribers of clinical 3D printed items; poor understanding of the impact of changing parameters has significant clinical impact.

## Conclusions

This study aimed to investigate the effect of post cure-protocol alongside print orientation, providing additional information to the literature by correlating spectral, colour and mechanical property data with post curing conditions. There was significant effect on tensile strength exhibited with varying post-cure parameters and print orientation. Temperature and time of post-cure impacted both mechanical and physical properties, with longer cure times and higher temperatures producing higher tensile strength, yet more yellow discolouration. This study provides novel insights in the mechanisms driving colour change and the mitigating impact of post-cure protocols on anisotropy which is critical for some applications. Dental restorations, for example, require careful consideration of both print orientation, post-cure conditions and aesthetics (such as colour); time and cost constraints in commercial laboratories may lead to suboptimal choices that compound material weakness and colour. Clinicians and researchers that take responsibility for the restoration must prescribe appropriate protocols equipped with background knowledge. Further standards, evidence-based guidelines and education as to the effects of printing protocols are required to ensure operators can achieve the desired mechanical and physical properties from printed products, maximising on the potential time, cost and material saving benefits in comparison to subtractive methods of manufacture.

## Data Availability

The datasets used and/or analysed during the current study are available from the corresponding author on reasonable request.
